# Ohio State Dental Law

**Published:** 1896-03

**Authors:** 


### EDITORIAL.

### Ohio State Dental Law.

A bill is now pending in the Legislature, the object of which is to amend or so change the present dental law as to make it more efficient. In the present law there are some deficiencies that render it almost valueless; at least, so far as it has been administered, it falls short of accomplishing the object for which it was enacted. In the new bill these deficiencies have been remedied, and certain very desirable features have been added.

The present bill is one that ought certainly, and we have no doubt will, command the respect and hearty support of every truly professional dentist in the State, and we see no good reason why it should not receive the hearty support of every well-wisher of our profession in the State.

There are some who are practicing in open violation of the law; these, of course, will oppose the passage of the bill. There are some, though I hope not a great many, who are so grossly ignorant that could not pass a reasonable examination before an examining board; these, of course, will oppose the passage of this bill. There are a few others who will oppose the bill from purely selfish motives—considerations that affect themselves and nobody else.

These are the oppositions that are to be met with by the friends of the bill, and it is to be hoped that the united effort of the better part of the profession in the State will be promptly put forth for the success of the measure.

